# Constipation: Its Causes, Consequences, and Rational Treatment1Read before the Buffalo Medical and Surgical Association, October 1, 1889.

**Published:** 1889-11

**Authors:** DeLancey Rochester

**Affiliations:** 469 Franklin street; Buffalo, N. Y.


### CONSTIPATION: ITS CAUSES, CONSEQUENCES, AND RATIONAL TREATMENT.[1]

By DeLANCEY ROCHESTER, M. D., Buffalo, N. Y.

I have chosen this so hackneyed a subject because I believe that a neglect to inquire into the state of a patient's bowels is a common cause of mistaken diagnosis, and, therefore, of mistaken treatment; and that if every one took pains to have a proper evacuation of the bowels every day, there would be far fewer cases of so-called general debility, nervous prostration, etc., that are so prevalent at present.

The causes of constipation are very numerous, but may be classed under three general heads, viz.: First—Conditions which obstruct the normal channel; Second—Too great dryness of the contents; Third—Variations in the functional activity of the motor apparatus, muscular or nervous, producing imperfect peristalsis.

Under the first head, conditions which obstruct the normal channel, we have (*a*) constriction of the gut from stricture, the result of former disease, such as dysentery, ulceration, colitis, etc.; (*b*) tumors, whether within the bowel or pressing on the bowel from outside; (*c*) volvulus; (*d*) invagination; (*e*) occlusion in hernial sac; (*f*) inflammatory exudations around gut; (*g*) congenital absence of anus.

These conditions are all of a more or less acute character, or are such that constipation is only one of the less prominent symptoms, and all require surgical interference for recovery. As that is not the class of cases which I wish to take up this evening, I will dismiss them without further remark.

The second condition mentioned—too great dryness of the contents of the bowel—is a very common cause of constipation; it may be due to (*a*) too little water in the diet, (*b*) diminution of digestive secretion, especially of the bile, or to (*c*) the excessive removal of fluid by other organs, as by the skin in profuse sweating, the kidneys in polyuria, whether of diabetic or other origin, or by the breasts in nursing mothers.

---

1. Read before the Buffalo Medical and Surgical Association, October 1, 1889.

The third condition—functional derangement of the motor apparatus—may be due to (*a*) affection of the chord or brain, in which case it is, of course, only one of the less prominent symptoms; or to (*b*) inflammations, degenerations, chronic catarrh, etc.; or to (*c*) lack of proper regular exercise of the parts.

I have scheduled the causes in this manner for purposes of ready reference in speaking of the remedial measures to be employed in overcoming the constipation.

However, before passing to that part of the subject, I wish to make a few remarks on some of the consequences of constipation.

Whatever the cause of the constipation, we have as the first result an accumulation of the fecal matter in the large intestine, from which the watery portions are rapidly absorbed, leaving hard scybalous masses to act as a source of irritation, or to decompose and produce poisonous materials for absorption into the circulation.

As a result of the presence of these hard scybalous masses in the rectum and colon, or of the absorption of poisonous gases or ptomaines resulting from their decomposition, there arise numerous and varied disturbances of the whole system.

First among these disturbances may be mentioned neuralgias of various parts of the body, more especially of the supra-orbital branch of the fifth cranial nerve. These cases are often accompanied by gastric disturbances, and are supposed to be due to indigestion, and a cathartic is given, followed by some form of pepsin or other digestive. The headache is relieved by the cathartic action, and the patient goes on for a longer or shorter period, and then comes down with another: the intervals between the attacks gradually grow shorter, until the patient believes that he or she must have a little headache all the time, and occasionally a very severe one. Thus life becomes a burden instead of a joy.

Next to the supra-orbital neuralgia, as a result of constipation, comes that feeling of general lassitude, heaviness, loss of appetite, loss of interest in what is going on, pains in the back, etc., etc., accompanied by a furred tongue and nasty taste in the mouth, more noticeable in the morning, which leads to the nervous prostration that is so fashionable at the present time.

Again, chronic constipation may cause a perversion of the secretions, thus producing gastric and intestinal indigestion, leading to a true anemia, which, reacting, increases the constipation, and is injured, rather than improved, by the administration of iron alone.

Disturbances of the menstrual function, and even chronic catarrhal endometritis, may sometimes be traced directly to constipation, and cannot be rectified until the constipation is overcome.

Even chronic bronchitis, with great emaciation, suggesting immediately phthisis, is sometimes due to obstinate constipation. I recall the case of a Mrs. G., who consulted my late father, some six or seven years ago. The history was that she had taken a severe cold, some years previously, that had settled in a cough, which she had been unable to shake off. She had steadily emaciated, and coughed severely, especially in the morning, raising considerable muco-pus. She had been under the care of several physicians, all of whom had told her she had consumption, and had put her upon cod liver oil. Still she did not improve. I have, unfortunately, forgotten the result of physical examination of the chest in this case, as it was merely related to me by my father to impress upon me the fact that all coughs with emaciation were not necessarily consumption.

Careful inquiry revealed the condition of obstinate constipation and dysmenorrhea, dependent upon, or at least accompanied by, posterior displacement of the womb. Correction of the displacement by means of a pessary, and the use of a bitter tonic and the regulation of the diet, overcame the constipation, the cough ceased entirely in less than four months, and the patient is now a healthy woman; after a lapse of nine years, she has begun bearing children again, having had two in the last six years.

Having referred thus briefly to the possible consequences of constipation, I will now call your attention to the methods of curing the same, which have proved beneficial in my hands.

In the first place, in asking a patient as to the state of his or her bowels, I am never satisfied with the statement that they are regular, but always ask specifically whether they move every day. It is surprising, especially in dispensary practice, to find the great prevalence of constipation. If you ask a dispensary case whether her (and it is a woman seven times out of nine) bowels are regular, she will say: "Well, they were regular to-day;" but when you ask her whether they moved the day before, she generally says they have not, and further inquiry reveals the fact that they have not moved in five, seven, nine, and, in one case that I had, fourteen days. In fact, these people make of their bowels miniature Hamburgh canals of stagnant, foul, decomposing material within their bodies, and then wonder that they have no appetite and that they feel so poorly.

But to return to the subject of treatment. A case of chronic constipation is before us; what shall we do for it? "Chronic constipation? Oh! yes, I can cure that. Give him So-and-so's anti-constipation pill of aloin, belladonna, and strychnine, gelatin-coated, made to suit all cases." Well, perhaps you may hit it right, and cure the patient; but that is not the proper way to treat such a case, and you may be doing a great deal of harm. We must remember that these three drugs are all powerful poisons, and especially that strychnine kept up too long may do serious harm to the nervous system.

A case in illustration:

H. F., a boy aged 16, consulted me for troublesome constipation producing, occasionally, piles, and always a painful movement. He had obtained a prescription from some other physician for pills of aloin, belladonna, and strychnine, which he had been taking, one or two at night, and one or two in the morning, for over a year. He was suffering from occasional intense headache; he awoke in the night with sudden jerks, and was troubled with nightmare. I examined him carefully. His tongue was clean, his appetite was good, except when he had headache, and he seemed in every way healthy, except for his constipation and the nervous state in which he was as a result of either the constipation or the pills.

On careful inquiry as to his mode of life and his diet, I found that he drank very little water. I told him to stop his pills, and to drink a tumblerful of cold water at night and one with his breakfast. The second day after, his bowels moved naturally, and continued to do so daily for a month, when he passed from under my observation.

Another case:

J. S., a boy aged 18, sent for me in a great hurry one night. I found him in great distress, from violent pain in the bowels, which had not moved in several days. The pain was so violent that I was forced to give him morphine hypodermically to quiet it.

I ordered his mother to give him a large enema of soap and water and sweet oil in the morning. The morphine quieted him, and the enema moved his bowels nicely. I then inquired into his condition, and found that he was a schoolboy, very fond of his books, that he did not take much exercise, and that, in consequence, his bowels had become more and more sluggish, until things had culminated in the attack for which I was called. Careful study of his case revealed no gastric or other digestive disturbance, but merely a sluggish condition of the bowel, with hard, painful evacuation, which occurred only once in three or four days.

For this case, I recommended regular out-of-door exercise and, temporarily, massage of the abdomen, rubbing the bowels in the direction of the colon, up on the right side, across, and down on the left side, for half an hour every evening and every morning. For two weeks, this treatment produced the desired result, and, as I have not seen him since, I judge the relief continues.

Another case:

A woman, between thirty and thirty-five years of age, consulted me in the Fitch Dispensary, complaining of loss of appetite, gastric pain when food was taken into the stomach, occasional vomiting, and neuralgic pains in various parts of the body. Further inquiry revealed the fact that she was constipated, never having a movement without taking "pills," which she would get at the drug-store. Her tongue was slightly coated. I gave her, to begin with, a dose of calomel, combined with the phosphate and bicarbonate of sodium. This was followed up by the use of a prescription, containing at a dose :

| | |
|---|---|
| Fl. ext. cascaræ sagradæ . . . . . . . . . . . . . . . . . . | M. x. |
| Tinc. nucis vomicæ . . . . . . . . . . . . . . . . . . . . . | M. x. |
| Tinc. cinchonæ comp. . . . . . . . . . . . . . . . . . . . . | ʒii. |
| Vini Xerici, q. s., ad. . . . . . . . . . . . . . . . . . . . | ℥ss. |

to be taken in water half an hour before each meal.

She has now been taking the medicine for a month. She has one good movement from her bowels daily, her tongue is clearing up, she no longer vomits, her appetite has improved, and she is feeling in every way better.

One more illustrative case, and then I will give you my general conclusions :

This case is a woman, of about forty-five years of age, who suffered from constipation for a number of years. She had been accustomed to a very sedentary life, and had accumulated considerable adipose tissue. She had a fair appetite, her tongue was not coated, she had no gastric distress, but she felt weak and unstrung, with occasional neuralgic pains in her face and side, and was markedly constipated, having a comfortable movement only by means of an enema.

She was given strychnine, gr. 1-50, three times daily before meals, was told to use her enemata for a week or two, and then stop their use. She did so, and continued to have movements a month after the enemata had been stopped, and the strychnine was being gradually reduced in amount when she passed from under observation.

From the study of quite a large number of cases of constipation, in both private and dispensary practice, I have come to the following conclusions, viz. : that the neglect of the important function of daily defecation leads to a poisoning of the system by absorption of gases and other materials, the products of decomposition of feces retained in the bowels ; that the nervous system is seriously affected directly by the poisons thus absorbed and, reflexly, by the hardened masses of feces in the rectum and colon ; that, as a result, neuralgias of various nerves make themselves felt ; that the digestive secretions are perverted, thus interfering with the proper nourishment of the individual, and leading to anemia and all its train of ills : that it is more often due to neglect upon the part of the individual to respond to the calls of nature at the proper time than to any other one cause, thus producing a habit of constipation. This is often the case in early childhood, and it is our duty, as physicians, to impress upon mothers the necessity of compelling one movement from the bowels of the baby and young child at a given hour every day. This can generally be accomplished by attention to diet, and by placing the child on the stool regularly at the same hour every day.

In every case of constipation that presents itself, it is our duty to study carefully the conditions and mode of life of the individual to learn the causes of the constipation and overcome it by hygienic means rather than by the administration of cathartics and laxatives. Many cases can be cured by the simple administration of cold water morning and evening. Where the constipation is due to the diminution of digestive secretions, this can be often overcome by proper exercise, such as rowing, horse-back riding, etc. Where exercise alone will not accomplish the result, the administration of some simple bitter tonic will excite the normal secretion and thus overcome the constipation ; when the simple bitter tonic will not accomplish this result, the use of some substance with the special property of increasing the flow of one or more of the gastro-intestinal juices will be beneficial. The use of senna or cascara sagrada in combination with nux vomica and cinchona, forms an excellent remedy. But for the accomplishment of the desired results, these medicines should not be given in cathartic doses, but in doses of from seven to fifteen minims, given *before* each meal, if possible, a half hour before.

After it has been taken for a week or two weeks, the dose should gradually be reduced, and finally the medicine abandoned altogether. In those cases in which gastric catarrh is one of the causal factors, or, at least, an accompaniment of the constipation, systematic lavage, or the sipping of half a pint of hot water one hour, or half an hour, before each meal, is generally followed by most excellent results.

In cases of anemia with constipation, I have obtained most gratifying results by the use of Carlsbad salts, given in doses of one-half or one teaspoonful in four or six ounces of hot water, half an hour before each meal, and carbonate of iron in the form of Blaud's pills or Vallet's mass, or tinct. ferri chloridi, after each meal. The administration of the iron in these cases, without the simultaneous use of a laxative, is worse than useless.

In cases in which a lack of tone of the lower bowel is the evident cause of the constipation, as in people who lead a very sedentary life, regular systematic massage of the abdomen for half an hour, morning and evening, rubbing in the direction of the course of the colon from the cecum to the rectum will generally bring about the desired result.

In those obstinate cases in which this mode of procedure is not successful, the administration of strychnine in doses of from 1-50 to 1-20 of a grain, three or four times a day, kept up for a considerable time, in addition to the massage, is generally all that is needed. It is seldom that it is necessary to add aloin to the strychnine, but when it is, it had better be combined with belladonna also, to prevent the griping, as in the aloin, belladonna and strychnine pill, which, we thus see, has its proper place—though a very limited one—in the treatment of chronic constipation.

The use of enemata, or of glycerine, or soap suppositories, is only palliative and produces no permanent benefit.

To recapitulate, the physician should never forget to inquire into the condition of his patient's bowels, remembering that lack of the proper performance of their function has far-reaching results of a direful character; and in the treatment of chronic constipation it should be his aim to search carefully for the cause and remove it by the use of *hygienic measures*, such as the liberal use of water, fruits in their season, and other dietary means; by regularity of habits, by proper out-of-door exercise, by massage of the abdomen.

The great majority of cases can be cured by the use of one of these measures, or a combination of several. When, however, they one or all fail, as occasionally they do, we may turn, as a last resort, to the proper use of appropriate medicines. There can be no iron-clad rule as to what one medicine is useful in chronic constipation. Each case must be carefully studied and treated according to its individual necessities. When it is necessary to use medicines in the treatment, it is well to begin with one good purge by the use of calomel, blue mass, or the compound cathartic pill of the U. S. P., to be followed by the systematic use of the remedies indicated in the special case, remembering that it is very seldom necessary to use a true cathartic in such cases, but generally a stimulant, either to the digestive secretions, or to the muscular or nervous motor apparatus.

469 FRANKLIN STREET.

